# Topical prebiotic nitrate: optimizing the ‘hang-time’, source and dose for specific oral or systemic effects

**DOI:** 10.1038/s41522-024-00528-2

**Published:** 2024-07-18

**Authors:** Bob T. Rosier, Alex Mira

**Affiliations:** Department of Genomics and Health, FISABIO Foundation, Valencia, Spain

**Keywords:** Applied microbiology, Dental conditions, Microbiome

## Abstract

In our opinion, the ‘hang-time’ of nitrate-containing products discussed in the letter by Green and Green is an interesting variable that should be considered when nitrate-based treatment or prevention strategies are designed. However, due to direct nitrate recycling after nitrate intake, products with a long ‘hang-time’ (e.g., chewing gum) may not always have an advantage compared to products with a short ‘hang-time’ (e.g., vegetable juices). We argue that extending the ‘hang-time’ is especially relevant and potentially beneficial for different applications, such as using a low nitrate dose to stimulate the oral effects, reaching oral tissues that may otherwise not be exposed to dietary nitrate (e.g., periodontal pockets), and providing a longer nitrate exposure in individuals with an impaired salivary flow. Apart from the ‘hang-time’, other important variables are the nitrate dose and source (e.g., different salts and vegetable extracts), as well as the desired effect (e.g., an oral effect versus systemic effects). Finally, we believe that the alterations in salivary microbiota observed before and after chewing three nitrate-rich gums over a period of ~5 h, as reported by Green and Green, could be considered beneficial. However, the oral microbiota composition is affected by the circadian rhythm and the effect of gum mastication should be evaluated. These results should thus be confirmed by a placebo-controlled study, where these confounding factors can be accounted for.

The nitrate-nitrite-nitric oxide pathway is a fascinating example of symbiosis between the human body and the oral microbiota, providing both oral^1^ and systemic^2,3^ health benefits. Apart from dietary nitrate from vegetables, this pathway could be stimulated with nitrate-containing products. Dietary nitrate increases salivary nitrate levels after ingestion and absorption through the gastrointestinal tract, and this increase can persist for more than 4 h^4^.

In our opinion, the ‘hang-time’ of nitrate-containing products discussed in the letter by Green and Green is an interesting variable that should be considered when nitrate-based treatment or prevention strategies are designed. A common idea is that it takes around 1 hour for swallowed dietary nitrate to reach the salivary gland after absorption through the intestine, starting the recycling cycle via the blood stream that keeps the salivary nitrate concentrations elevated for several hours. This would imply that a benefit of a nitrate chewing gum would be to increase the nitrate between the initial intake and the first recycling – a period in which salivary clearance would normally eliminate all nutrients. However, some data indicates that after intake of liquid nitrate supplements (e.g., beetroot juice or vegetable extracts dissolved in water), which are assumed to have a short ‘hang-time’, the nitrate clearance by saliva is not as it could be expected. For instance, current literature^5,6^ and our preliminary data (Fig. 1) indicate that liquid nitrate supplements cause an unsuspected increase in the first 10–30 min right after intake. The salivary nitrate dynamics in the first 30 min are in contrast with other components (e.g., fluoride)^7^ or nutrients (e.g., sugar)^8^, which decreases over time right after intake until they disappear from saliva. For example, after a sugar-rich fruit juice, salivary sugar increases right after intake, but after 3 min most sugar (>90%) has been eliminated followed by complete clearance in 10–30 min^8^. The direct increase of salivary nitrate after intake could be due to oral biofilms and tissues retaining nitrate more efficiently than other nutrients, vegetable particles trapped in the mouth being degraded and releasing nitrate, nitrate reaching the intestine before other nutrients and/or nitrate recycling starting before reaching the intestine. The latter could well be possible as the oral tissue is highly vascularized and directly connected to the bloodstream.

**Fig. 1:**
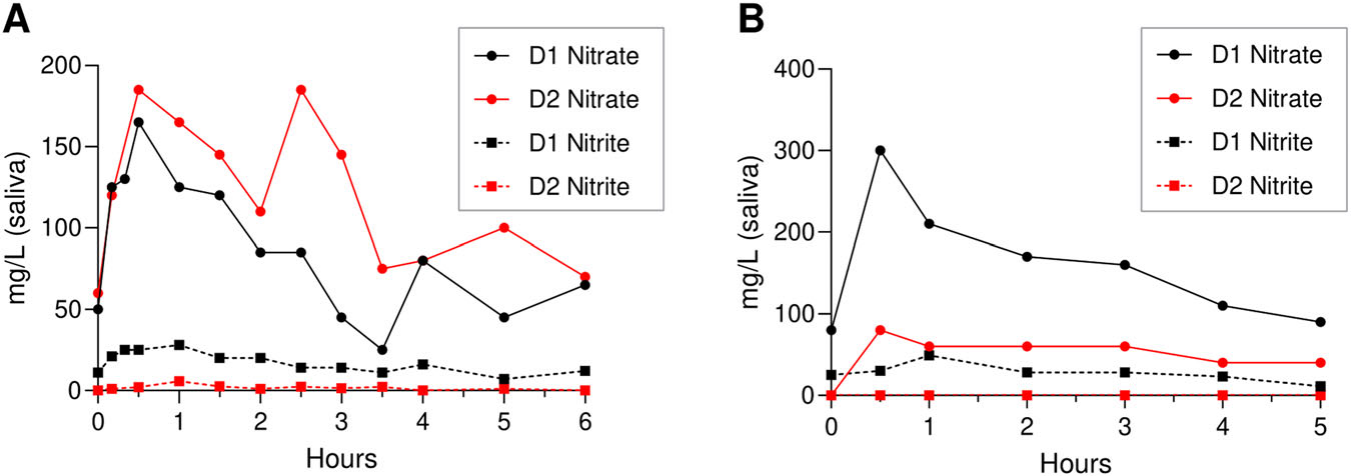
Salivary nitrate and nitrite of two donors after consuming liquid beetroot supplements. Doses of RedNite (**A**) and NutriSpain (**B**) beetroot extracts containing 220 mg nitrate (according to the nitrate content provided by the manufacturers) were dissolved in 200 ml water. Two different donors donated saliva before supplement intake (0 h) and then consumed the supplement within 1 min. The RedNite (**A**) and NutriSpain (**B**) experiments were performed on two different days with the same two adult male donors. **A**: the salivary nitrate concentrations show an unexpected increase between 10 and 30 min after supplement intake, reaching similar levels in both donors. Donor 1 appears to reduce more nitrate to nitrite, while donor 2 shows a second peak after 3 h, resulting from nitrate recycling. **B**: 30 min after supplement intake, the salivary nitrate of donor 1 is more than three times higher than the salivary nitrate of donor 2. On this day, it appears that nitrate recycling of donor 1 is more efficient than donor 2 during the entire period of 5 h. Additionally, no nitrite production was detected in the saliva of donor 2. This data indicates that there can be inter- and intra-individual differences in the nitrate recycling and reduction when consuming the same nitrate dose. Donors were requested to avoid vegetables, fruits, and processed meats for breakfast at least 1 h prior to donating the 0 h sample. Salivary nitrate and nitrite measurements were performed as described previously^17^. The increase in salivary nitrate between 10 and 30 min after nitrate intake were also observed by Subramanian and Gupta (2016)^5^ and Lundberg and Govoni (2004)^6^ in populations of 16 and 9 individuals, respectively.

Subramanian and Gupta (2016)^5^ gave a nitrate-rich amaranth extract dissolved in water to 16 individuals and show that there is an initial increase of salivary nitrate in the first 15–30 min after intake. Then, a large peak is formed and maintained between 30 min and 3 h with the highest levels at 2.5 h^5^, which are thought to result from nitrate being recycled after absorption through the intestine. The salivary nitrate concentration clearly decreases between 3 and 8 h but stays above baseline levels. The effect of chewing gums containing similar doses of nitrate on these dynamics are unknown, and Green and Green did not provide salivary nitrate measurements after the use of three nitrate chewing gums, each containing 0.62 mmol (38.4 mg) nitrate (i.e., a total of 1.86 mmol or 115.3 mg nitrate), over a period of ~5 h. If a significant portion of nitrate, nitrite and/or nitric oxide directly enters the blood stream via the highly vascularized oral tissues, nitrate-containing chewing gum could lead to more efficient nitrate recycling during the first hour after intake. Thus, the dynamics of nitrate recycling and availability in the oral cavity after the use of chewing gum, as well as after dentifrice use, should be explored in future studies.

We argue that extending the ‘hang-time’ is especially relevant and potentially beneficial for different applications, such as using a low nitrate dose to stimulate the oral effects, reaching oral tissues that may otherwise not be exposed to dietary nitrate (e.g., periodontal pockets), and providing a longer nitrate exposure in individuals with an impaired salivary flow. Apart from the ‘hang-time’, other important variables are the nitrate dose and source (e.g., different salts and vegetable extracts), as well as the desired effect (e.g., an oral effect versus systemic effects).

Regarding nitrate amounts, to lower blood pressure, doses of >200 mg (on average 500 mg) in ≥ 70ml of liquid nitrate supplements (mostly beetroot juice) have been used in different studies (reviewed by Ashworth and Bescos^9^). Similarly, to increase sport performance, the doses are usually >250 mg (up to 1000 mg) (reviewed by Shannon et al.^10^ and Macuh and Knap^11^). Taking into account the Acceptable Daily Intake (ADI) of 3.7 mg nitrate per kg bodyweight (222 for an adult of 60 kg), these doses can be considered high. After consuming a high dose of nitrate with a short ‘hang-time’, an increase in systemic nitric oxide levels results in the desired effect 1.5–3 h after intake when sufficient nitrate has been recycled and converted into nitrite and nitric oxide by the oral microbiota. It should be explored if products with a longer ‘hang-time’, such as a chewing gum, could lower the dose or the time until the desired effects. Additionally, the safety of concentrated high nitrate doses should be studied as N-nitroso compounds could be formed in acidic oral biofilms, which could be limited by antioxidants naturally found in saliva or added to the products^1^.

Another thing to keep in mind, if a chewing gum would need to contain 200 mg nitrate to obtain the desired systemic effect, this could temporarily lead to non-physiological high nitrate concentrations. Natural concentrations after vegetable consumption are often between 5 and 8 mM, but 10–20 mg of nitrate (5–10% of 200 mg or ~25–50% of the 38.4 mg nitrate used by Green and Green) released from a chewing gum in 1 ml of saliva leads to a concentration of 161.3–322.6 mM. Such high nitrate concentrations can directly inhibit oral bacteria or be converted to high nitrite and nitric oxide levels with inhibitory effects. For example, in an in vitro study, *Steptococcus mutans* was inhibited by 100 mM nitrate but already by 0.5 mM nitrite, while *Veillonella atypica* and *V. parvula* were inhibited by 20 mM nitrite but not by 100 mM nitrate or 0.5 mM nitrite^12^. The antibacterial effects of nitrate resulting from high dose inhibition are not the same as the prebiotic effect of physiological concentrations of nitrate, which, as stated by Green and Green, have been shown to increase eubiosis and prevent acidification when incubating saliva in sugar-containing growth medium^13,14^. Theoretically, if high doses of nitrate and nitrite saturate the nitrate- and nitrite-reductases, the reduction processes are blocked, which could hamper the associated benefits. We therefore suggest that the local nitrate dose delivered by chewing gum should be adjusted to maximise the desired beneficial effects.

In our originally in vitro study^15^ to which Green and Green replied, incubating subgingival plaque of periodontitis patients with 50 mM nitrate during 12 h provided additional benefits compared with 7 h incubation with 5 mM nitrate (both achieving a ~ 50% of nitrate reduction of each concentration). This indicates that a periodontal gel with a dose of nitrate above physiological levels could lead to additional benefits compared with a physiological concentration. However, the studied parameters were microbial composition and biofilm growth of dysbiotic subgingival plaque in an in vitro system. It should be tested if the desired effect is maintained in vivo and if other desired oral effects (e.g., limiting acidification of supragingival plaque) could also benefit from doses above the physiological level and to what extent. Importantly, concentrations of 5-50 mM nitrate are obtained by adding only 0.31–3.10 mg of nitrate to 1 ml periodontal gel. Similarly, to obtain post-vegetable physiological concentrations of 5–8 mM, only 0.31–0.51 mg needs to be released into 1 ml saliva. Topical products with a longer ‘hang-time’, such as chewing gums, chewing tablets or gummies, could thus gradually release low amounts of nitrate to stimulate the oral benefits. This could be achieved with doses that are much lower than 200 mg (commonly used to stimulate the systemic effect of nitrate), and conceivably also lower than 38.4 mg (used by Green and Green), limiting potential safety concerns and/or high salivary nitrate concentrations with undesirable inhibitory effects.

By comparing the salivary microbiota before and after chewing three chewing gums (each containing 38.4 mg nitrate, leading to a total intake of 115.3 mg nitrate) over a period of ~5 h, Green and Green found significant changes. Specifically, these were an increase in health-associated *Neisseria flavescens* and *N. subflava*, and a decrease in *Prevotella melaninogenica* and periodontitis-associated *Fusobacterium periodonticum*, *F. nucleatum* and *Parviromonas micra*. While these changes could be considered beneficial, it is known that the oral microbiota composition is affected by the circadian rhythm, including nitrate-reducing species and periodontitis-associated bacteria. For example, *Neisseria* and *Fusobacterium* levels have been shown to fluctuate over the hours of the day^16^, while *Rothia* species can increase 4 h after water intake in the morning^17^. Additionally, the effect of masticating the three gums should be evaluated as this can affect plaque quantity, saliva production and oral microbiota composition. The results by Green and Green should thus be confirmed by a placebo-controlled study where these confounding factors could be accounted for.

Apart from the nitrate dose, another relevant variable is the nitrate source. As we previously discussed^1^, current potassium toothpastes (for gum sensitivity) often contain 5% potassium nitrate, which results in a dose of 30.7 mg of nitrate in 1 g of toothpaste (without antioxidants). Apart from delivering a high dose, potassium has been associated with periodontal dysbiosis^18^, so studies exploring the effect of different concentrations of this salt, or the potential use of alternative nitrate salts should be performed. Another option would be provided by vegetable extracts, which naturally contain a variety of antioxidants that could stimulate nitrate-derived nitric oxide formation and prevent the generation of N-nitroso compounds, but the presence of pigments that could stain the teeth should be evaluated. In our current^15^ and previous in vitro studies^13,14^, we used 5–50 mM sodium nitrate, finding different prebiotic effects. Thus, the consistency of these effects when changing the nitrate source should be explored.

Another benefit of products with a long ‘hang-time’ is that these could reach oral tissues that are not exposed continuously to saliva, such as communities inside deep periodontal pockets. This could be one of the reasons why the proportion of health-associated *Rothia* and *Neisseria*, which are genera that benefit from salivary nitrate, decrease in periodontal pockets. Periodontal gels with nitrate and possible other components that promote nitrate reduction and/or nitric oxide formation (e.g., antioxidants or selected carbon sources) could stimulate the growth of health-associated nitrate-reducing bacteria. Similarly, to increase nitrate exposure of the upper jaw and interproximal surfaces, a nitrate-containing varnish could be applied, and other nitrate delivering vehicles could be envisaged for specific purposes.

It is important to note that oral and systemic effects do not always go hand in hand, as a consequence of different timings or different optimal dosing. Data in Fig. 2, for instance, show a significant pH buffering effect of nitrate 2 h after intake, with the absence of a significant effect on blood pressure. In fact, strategies that specifically activate a given metabolic pathway could be envisaged, like denitrification to nitric oxide for antimicrobial or systemic effects, or nitrite reduction to ammonium (during which most protons are consumed^1^) for pH-buffering and caries prevention. In this regard, several environmental conditions, including pH and the protein:carbon ratio^19^, have been shown to influence these alternative metabolic routes and therefore the formulation of a nitrate-delivering product could be tailored for specific purposes.

**Fig. 2:**
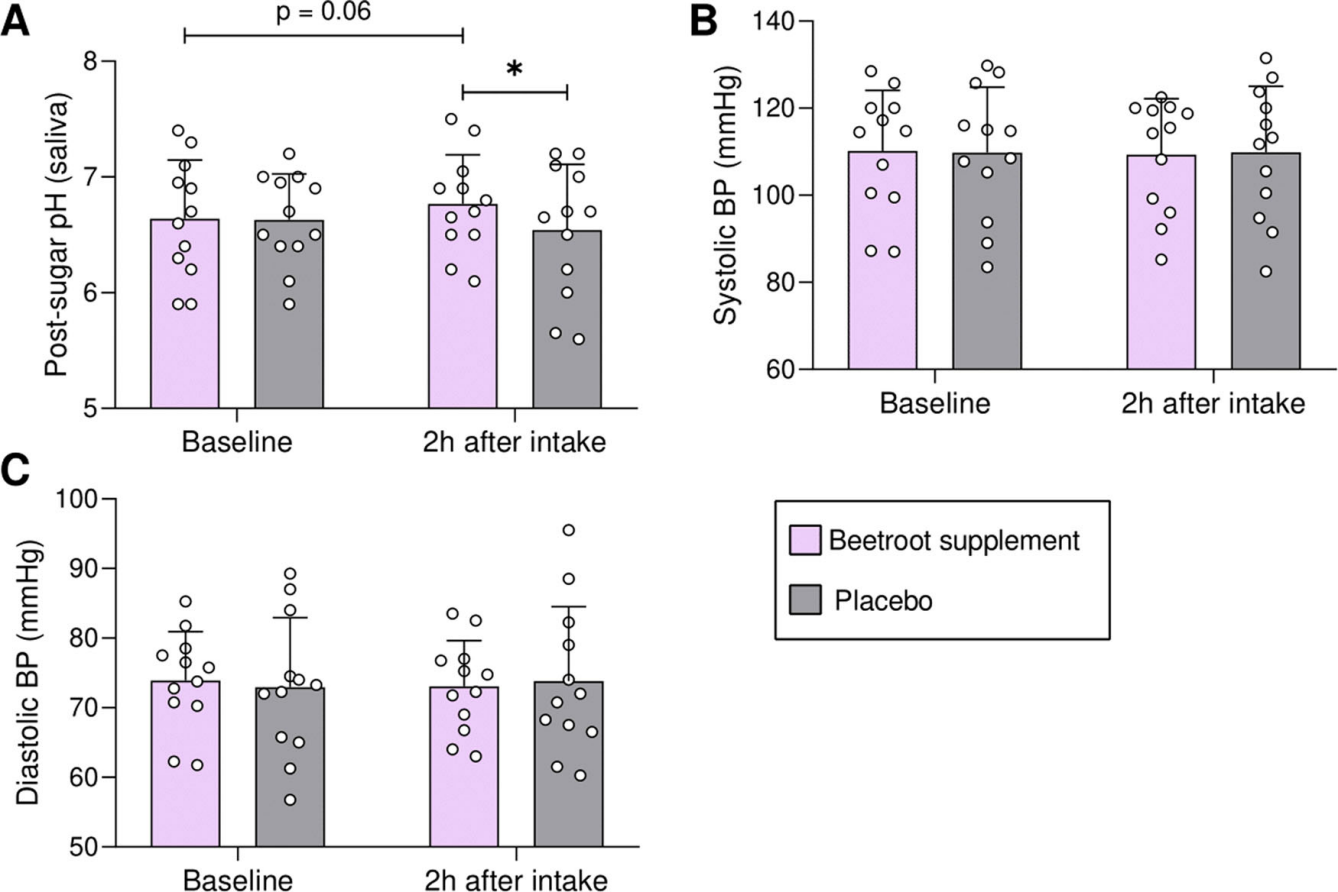
A nitrate-rich beetroot supplement limited oral acidification but did not affect blood pressure. In Rosier et al. (2021)^17^, 12 individuals received a nitrate-rich beetroot supplement, which was compared to a placebo in a blinded crossover setting. In this graph different parameters are shown before (baseline) and 2 h after intake of a nitrate-rich beetroot supplement (250 mg nitrate per dose according to the nitrate content provided by the manufacturer) (purple bars) or a nitrate-poor orange placebo (< 6 mg nitrate per dose) (grey bars). In (**A**), the pH after sugar exposure (10 min after a sugar rinse) was significantly higher after nitrate supplement intake compared with placebo supplement intake. This shows that nitrate limits oral acidification. However, no effect on systolic (**B**) and diastolic (**C**) blood pressure was observed. The pH data was published previously^17^, while the blood pressure was not. In short, individuals were instructed to sit down for 5 min and blood pressure was measured with an Automatic Blood Pressure Monitor Model M6 Comfort IT (OMRON Healthcare Europe B.V., Hoofddorp, Netherlands) twice on each arm.

Finally, a key point that needs to be addressed is the inter-individual variability in the systemic nitrate recycling and metabolism. Our own preliminary data in two individuals (Fig. 1) and current literature in larger populations^4,17,20,21^ indicate that individuals can show clear differences in the salivary nitrate concentration reached after the ingestion of a nitrate, as well as the amount of nitrate reduced to nitrite. The consequence of having a low or high nitrate recycling or reduction capacity for oral and systemic health should be evaluated, as this could identify individuals at special need of nitrate supplementation, ranging from Sjögren patients to individuals on medications that reduce salivary flow. Furthermore, the addition of nitrate-reducing probiotics to products with a longer ‘hang-time’ could stimulate nitrate metabolism and provide additional benefits.

In conclusion, we share the opinion of Green and Green that virtually all clinical studies available have been performed using vegetable supplements as nitrate source, and other alternatives must be evaluated and tested to enhance the effects and bioavailability of nitrate. However, we believe that various factors have to be evaluated to determine the optimal nitrate-delivering vehicles, sources, and dosages that, taking advantage of the remarkable coadaptation of oral microbes and human physiology, may contribute to disruptive and promising strategies to promote specific oral or systemic health parameters. Additionally, we think that the salivary nitrate and nitrite dynamics in the study presented by Green and Green should be determined, and the effects on the oral microbiota composition should be confirmed in a placebo-controlled study to account for confounding factors.
